# There’s More to Humanity Than Meets the Eye: Differences in Gaze Behavior Toward Women and Gynoid Robots

**DOI:** 10.3389/fpsyg.2019.00693

**Published:** 2019-04-24

**Authors:** Jessica M. Szczuka, Nicole C. Krämer

**Affiliations:** Social Psychology: Media and Communication, University of Duisburg-Essen, Duisburg, Germany

**Keywords:** human–robot interaction, sex robots, eye tracking, visual attention, human likeness

## Abstract

Based on evolutionary psychological theories, numerous eye-tracking studies have demonstrated how people visually perceive a potential mate in order to efficiently estimate the person’s mate value. Companies are currently working on sexualized robots that provide numerous human-like visual cues which foster the visual resemblance to humans. To gain more elaborated knowledge on how people react to sexualized robots compared with humans, the present study empirically investigated whether heterosexual males transfer deep-rooted evolutionary psychological processes of mate perception to human-like and machine-like sexualized robots. Moreover, we aimed to learn more about the processes of orienting responses toward human and non-human stimuli and about potential predictors of visual attention to robots. Therefore, we conducted an eye-tracking study in which 15 heterosexual men, 12 homosexual men, and 18 heterosexual women were confronted with stimuli showing women, human-like gynoid robots and machine-like gynoid robots. For the sample as a whole, there was no difference in the amount of time spent looking at the human and non-human breasts. However, the results for the heterosexual males supported the assumption that human breasts attract more visual attention than do the breast areas of human-like and machine-like robots. The pelvic region yielded an unexpected gaze pattern, as all participants spent more time looking at the robotic pelvic area than at the human one, with more visual attention paid to the machine-like robots than to the human-like robots. The results of the viewing times toward the head revealed that all participants had a stronger need to gain visual information about the human head in comparison to the robotic heads, underlining the importance of authenticity in terms of emotions and motivations that can only be decoded in humans. Moreover, the study showed that individuals more frequently switched their visual attention toward different body parts of the robots in comparison to the female stimuli, implying that non-human sexualized representations evoked a higher need for visual exploration.

## Introduction

The statement “Mating is a human universal” (Buss and Schmitt, 1993) not only implies that almost every human being, due to hormonal events and sociocultural influences, starts to develop sexual interest in women or men during adolescence (Miller and Benson, 1999), but also emphasizes the importance of mating for reproduction and evolution. To enable optimally successful reproduction, humans have therefore developed strategies to effectively estimate a person’s mate quality by looking at information provided by the person’s body (Buss, 1999). Consequently, studying eye movement patterns when people are looking at a potential mate has become an important research area in the field of human mating behavior. Results of numerous eye-tracking studies with heterosexual men highlight the visual importance of the female head, the chest and the pelvic region, since these body parts provide information about a woman’s reproductive value (e.g., Nummenmaa et al., 2012).

While the shape of the female body has been of aesthetic interest for centuries, it now also serves as a paragon for robots. Robots are machines which are built for specific reasons, ranging from work tasks in which they act autonomously and without human contact (e.g., industrial robots or robots which inspect and defuse explosive devices) to fields of application in which robots are built to have physiological and psychological contact with humans (e.g., robots in healthcare; Dautenhahn, 2007). In line with technological developments that are shaped to fulfill sexual needs (Allen, 2000) for example internet applications, VR technology), there have also been first attempts to develop robots that engage in intimate interactions with users. Companies (such as Abyss Creations/Realbotix) are currently developing human-like robots that are made to fulfill sexual needs by equipping hyper-realistic sex dolls with motors for movement and with a database providing the possibility to interact verbally. In his seminal book, David Levy (2008) predicted that by 2050, people will not only have sex with robots on a regular basis, but will also have relationships with and even marry robots. Levy highlighted that a prerequisite for these developments is that robots are “like us” with regard to their behavior and appearance.

This raises the question of whether people will use the same deep-rooted evolutionary psychological mechanisms of mating with a robotic replication of a human being, or whether mating with a robot will lead to new forms of information processing. As processes of visual attention have been shown to be influenced by the way people perceive a potential partner (e.g., Buss, 1999; Nummenmaa et al., 2012; see section “Visual Information on the Female Body and Its Perception” for details), we aimed to empirically investigate processes of visual attention to women and to female-looking. robots. According to evolutionary psychological approaches, it would be useless to process the human body and the mechanical body in the same way, because a mechanical body does not provide any authentic information about health, age, motivation, emotion, and consequently reproductive value (see section “Visual Information on the Female Body and Its Perception” for details). However, given that the mechanisms of mating are deeply rooted in humans (Buss, 1999), it is conceivable that the female-looking shape of the robotic female body is sufficient to trigger unconscious processes of visual attention that are similar to the way men look at women.

Based on these considerations, the present study asks whether heterosexual men apply the same gaze behavior toward female-looking robots as toward women. The implicit measurement of eye tracking enables unbiased insights into the visual attention toward women and sexualized robots, as it is not influenced by social desirability, which is particularly important given that the theme of sex robots might lead to biased reactions. Garza et al. (2016), who themselves conducted an eye-tracking study on the perception of the female body, highlight why eye tracking is useful in this research area by stating that “Eye movements provide a ‘true’ measure because they reflect ongoing mental processes in real time, whereas ratings are typically ‘*post hoc’* processes that rely more on problem solving strategies” (p. 14). Moreover, we are interested in whether the robots’ appearance, in terms of how human-like or machine-like they look, influences gaze patterns. Additionally, we examined whether this would influence people’s visual attention insofar as unusual aspects would lead to more detailed exploration. As sexual interactions with inanimate objects like robots deviate from sexual norms (Abramson and Pinkerton, 1995; Worthen, 2016), responses to explicit measurements are likely to be influenced by social desirability and reflections on societal and sexual norm adherence. This problem can be avoided by using eye tracking to measure eye movements, as studies have found that eye movements quantify visual attention without an influence of social desirability. For instance, an empirical study by Fromberger et al. (2012) indicated that it is possible to determine a person’s sexual orientation based on gaze pattern, which in the case of explicit measurements may also be influenced by processes of social desirability. The present study therefore aims to contribute unbiased empirical data regarding men’s initial reactions to robotic replications of women. To further scrutinize whether evolutionary aspects play a role, in the sense that gaze patterns are caused by the unconscious intention to check for mate value, we included heterosexual women and homosexual men as control groups. Furthermore, we assessed different personality traits as well as participants’ evaluations of the robots (including ratings of attractiveness) in order to examine their potential explanatory power regarding the amount of time participants spend looking at different body regions of the robots. Overall, the present study aims to provide further insights into how sexualized robots are perceived in contrast to women. As such, the work contribute to the discussion on sexualized robots, which has so far primarily been based on the work of scholars who discuss normative questions (Levy, 2008; Richardson, 2016).

## Literature Review

### Visual Information on the Female Body and Its Perception

When a person sees a potential mate for the first time, the body already provides information which is useful for estimating his or her mate value. Following an evolutionary psychological perspective, men are attracted to certain body features that indicate a good reproductive value of women. This value is influenced by both the health and age of a women, for which the female body provides observable information (Symons, 1995; Buss, 1999). The visual attention toward different body parts can by quantified by measuring a person’s gaze behavior. Here, eye tracking is a useful method to investigate the relative importance of different visual areas (Holmqvist et al., 2011). Combined with the fact that the technology is developing quickly and providing ever more precise data, eye tracking has become a popular method for research in the field of mating and attractiveness. In an eye-tracking experiment, Fromberger et al. (2012) empirically demonstrated that humans are visually drawn to sexually relevant stimuli when they are simultaneously confronted with an additional non-preferred stimulus. The authors concluded that the visual attention toward “evolutionary meaningful” information is deeply rooted. The following sections therefore explain evolutionary psychological mechanisms which drive males’ attention to different visual signals provided by the female body in order to efficiently estimate a woman’s potential reproductive value.

#### Torso: Waist-to-Hip Ratio and Breasts

With the onset of puberty, the body shape begins to transform. Caused by estrogen and testosterone, the sexually dimorphic body fat distribution leads to visual differences in the body shapes of men and women. During this process, women not only grow breasts, but also gain fat deposits on their buttocks (including upper thighs) and hips. The “gynoid” fat distribution, which is visually associated with the shape of an hourglass, can be quantified as waist-to-hip ratio (WHR, ratio between the size of the waist and hips) (Singh, 1993; Buss, 1999; Singh and Singh, 2006; Swami and Furnham, 2008). Different researchers (e.g., Symons, 1995; Singh and Singh, 2006) argue that a woman’s body shape helps to provide an efficient estimation of her mate value. More specifically, they state that visual cues can provide information about a woman’s age and health and therefore her reproductive value (Symons, 1995; Buss, 1999). Based on empirical studies, Singh (1993) hypothesized that men use the WHR as a visual “first-pass filter, which would automatically exclude women who are unhealthy or who have low reproductive capacity” (p. 304), without being aware of this initial selection process. The visual sign of health and reproductivity (represented by a WHR of 0.7 and lower) was empirically shown to be connected to ratings of attractiveness (Singh, 1993; Henss, 2000).

Another important part of the female body is the breasts, which are located in the upper ventral region of the torso. For females, the breasts not only play an important role in biological processes (e.g., breastfeeding offspring) but are also strongly associated with gender identity. Millsted and Frith (2003) concluded in this regard that “Breasts are seen simultaneously as a marker of womanhood, as a visual signifier of female sexualization, as synonymous with femininity, and as essential for the nurturance of infants” (p. 455). Regarding their visual importance in the process of mate selection, Marlowe (1998) postulated the nubility hypothesis, which states that growing breasts signalize sexual maturity, as they are a result of the fat distribution during puberty. Moreover, he argues that breasts are an honest signal of youth, as their shape changes over the lifespan.

Numerous eye-tracking studies have provided empirical evidence that the breasts and hips (as part of the WHR) of women are important visual sources of information (Dixson et al., 2011; Garza et al., 2016). However, various eye-tracking studies have also underlined that the visual importance of the body regions is linked to the task on which the participants are focusing and the explicitness of the stimuli. Bolmont et al. (2017) found that the body becomes a more important visual source of information when judging the sexual desirability of a women. The authors reported that under this condition, the chest was looked at for longer than the face. Gervais et al. (2013) found that when participants were asked to focus on a woman’s appearance rather than on her personality, they tended to look longer at the breasts and waist. This change in visual pattern can also be found when the explicitness of the stimuli is varied. As mentioned above, Nummenmaa et al. (2012) recorded the viewing behavior of participants who looked at naked and clothed full-body stimuli. Lykins et al. (2006) performed a similar study, showing erotic and non-erotic stimuli. The results of the two studies revealed a similar pattern: even though the faces were still the area which was fixated first and longest, the effect decreased when the stimuli showed erotic content or naked people. All in all, the aforementioned studies demonstrate that the female torso provides relevant visual information for men. Nevertheless, findings also suggest that visual importance is influenced by the explicitness of the stimuli or by the participants’ task. The more closely these aspects are related to reproduction (e.g., focusing on the sexual desirability of a stimulus or showing naked stimuli), the more important the information provided by the body becomes in comparison to the head.

#### Head

The female head has multiple attributes that provide information about a woman’s age, health, and emotional state, which in turn are all linked to her estimated mate value and therefore gain visual attention from heterosexual male observers. With regard to health, for instance, the brightness and color of the eyes (especially of the sclera; Gangestad et al., 2006), provide visual information which may be of visual interest to men. Further signs of age include pronounced lips and cheekbones, as they become more prominent during puberty (Johnston and Franklin, 1993). However, while some of the information mentioned above is probably more important in terms of mating, the head also conveys visual information relevant for all potential interaction partners, as it provides information about a person’s emotional and motivational state. In this respect, the eyes, along with the eyebrows and eyelids, are an important source of non-verbal communication (Kleinke, 1986). Such information is crucial, as it allows a potential interaction partner to decode emotions which are likely to correlate with a person’s inner state (emotions and intentions; Ekman et al., 1972; Darwin, 2000). The perception of the face is consequently deeply rooted, as it can be helpful, for instance, in order to detect a threatening or angry face and thus protect oneself from harm (e.g., Ohman et al., 2001). An eye-tracking study empirically confirmed the so-called “face in the crowd effect,” showing that if people are confronted with faces with happy and angry/ threatening expressions, they look more quickly toward the faces with the negative impressions (Shasteen et al., 2014).

A large number of eye-tracking studies have empirically confirmed the visual importance of the head even when people are confronted with the whole body of a person. The initial fixations of both male and female participants were found to be primarily on the face of both men and women, independent of participants’ gender (Hewig et al., 2008; Bolmont et al., 2014). This finding was also confirmed in a study investigating the gaze behavior while viewing pornographic videos, with participants showing an enhanced visual attention toward the face of the actress (Tsujimura et al., 2009). Nummenmaa et al. (2012) conducted a study in which they presented pictures of clothed and nude bodies to participants while recording gaze behavior. The results showed that even if the presented bodies were naked, the face was still the first source of information. Hassebrauck (1998) used a visual process method to find empirical evidence for the importance of the face as a source of information. Using a paradigm in which the participants got to choose which details of a full body image they wanted to uncover regardless of the order, it was found that the face (especially the eyes) was the most important source of information, as it was uncovered earlier than other body parts.

### Female-Looking (Sexualized) Robots

Robots that are supposed to be perceived as female are referred to as gynoid robots (Bar-Cohen and Hanson, 2009). While some researchers argue that robots do not necessarily need to be associated with a gender role (e.g., Haraway, 2006), others believe that it may be difficult to build genderless robots, as robots follow their designer’s idea of how a robot should look and act in order to perform a specific task (Søraa, 2017). Trovato et al. (2018) could show that especially the design of the shoulders and waist-to-hip ratio (WHR) do influence how strong a robot is associated with one particular gender. Parallel to the visual importance of the WHR among females (as it represents a visual sign for age/fertility as it starts to transform with the onset of puberty, see section “Torso: Waist-to-Hip Ratio and Breasts” for details), the study could show that a lower WHR and therefore the imitation of the hourglass shape caused more participants to assign the robots to the female gender. In fact, the majority of gendered humanoid robots are female in appearance (Alesich and Rigby, 2017). This is in line with current developments regarding sexualized robots, as 80% of the hyper-realistic sex dolls (which can be understood as a precursor of sex robots) of the company Realdoll have a female appearance (Bartneck and McMullen, 2018). In the case of these dolls, but also with regard to gynoid human-like robots, this means that the synthetic body is equipped with skin and details that contribute to the resemblance to a human being. Such details range from features that one may notice at first glance (fingernails, eyes, or hair) to properties that might be covered under clothing and may only be useful for specific sex applications, such as artificial vaginas. The visual information provided by sexualized human-like robots is therefore a replication of what humans have until now only encountered in their own species. Szczuka and Krämer (2017) showed that there is no difference between women and female-looking robots in terms of the associative strength of the concept of attractiveness. In other words, their experiment showed no differences in men’s reaction time when they were asked to assign words associated with the concept of attractiveness if they were primed with stimuli showing women compared to primes displaying female-looking robots with salient artificial body parts. The authors argued that the strength of the human-like cues, meaning the visual similarity to humans, may explain this lack of difference in reaction times. They further highlighted the potential importance of visual cues with the help of evolutionary psychological mechanisms, by arguing that men have a predisposition to be drawn to and react to visual cues that represent signs of reproduction and health. These human-like cues, combined with the fact that robots can engage in an interactive interaction, communicate in natural language and represent a social role which would normally be assumed by a human (for instance, in the case of sexualized robots a (sexual) interaction partner), are likely to evoke mindless social reactions to the machines (Nass and Moon, 2000). This process is reflected in the media equation theory of Reeves and Nass (1996), who summarize that “when our brains automatically respond socially and naturally because of the characteristics of media or the situations in which they are used, there is often little to remind us that the experience is unreal. Absent a significant warning that we’ve been fooled, our old brains hold sway and we accept media as real people and places” (Reeves and Nass, 1996, p. 12). To name just a few examples of how these social reactions manifest themselves, studies have revealed that if confronted with a stimulus that provides a sufficient number of social cues, people show reactions of politeness toward computers (Nass et al., 1999), or that machines are able to evoke feelings (e.g., Bartneck et al., 2010). In the context of the present study, it is important to highlight that all of these reactions are accompanied by processes of visual perception. Transferred to sexualized female-looking robots, it is therefore conceivable that heterosexual males will initially approach the robotic replications in the same way they would react to women, including similar processes of visual perception.

With regard to potential users, there are individuals who deliberately engage in a romantic or sexual relationship with a non-living entity for reasons such as the possibility to act out sexual preferences without the fear of being judged (Danaher et al., 2017) or social deficits (Szczuka and Krämer, 2017). Contrary, there are also reasons to believe that people may avoid the technology. Besides the deviation from societal and sexual norms (Worthen, 2016), MacDorman and Ishiguro (2006) named the deeply rooted avoidance of genetically inadequate partners as one of the reasons for the aversion which some individuals might have to human-like robots. Sexualized robots therefore have the potential to evoke contradictory responses of both avoidance and approach (Lewin, 1935), which are likely to manifest themselves in the way people perceive robots (e.g., by gaining visual information).

### The Present Study

Throughout evolution, people have developed strategies to efficiently estimate different parameters which are of importance regarding interpersonal processes of mating, such as age, health, and emotional and motivational states, by looking at specific visual cues of the body. Given that human-like, sexualized robots will soon be commercially available, it needs to be asked whether people will apply these deeply rooted mechanisms to robots, or whether they will instantly reflect on the fact that the visual information of the robots does not provide any authentic information. Based on the assumptions of the media equation theory, one could argue that people mindlessly react to robots by activating a “social script.” This could contribute to provoking an initial reaction to sexualized robots that is similar to the initial reaction to humans, due to their similar physique. Although there are studies demonstrating people’s initial visual reactions to the bodies of persons (see section “Visual Information on the Female Body and Its Perception” for details), to our knowledge, no eye-tracking study has yet presented participants with robots or visual stimuli showing robots in contrast to humans in order to investigate differences between the two stimuli regarding initial visual perception. As 80% of the purchasers of hyper-realistic sexualized dolls (which currently represent the precursor of sexualized robots) are males, who mostly buy female-looking sex dolls (Bartneck and McMullen, 2018), we were particularly interested in the question of whether the main target group (heterosexual men) would show different gaze patterns when looking at women and robots. However, we also included heterosexual women and homosexual men as control groups. This should help to sufficiently distinguish the gaze behavior of the heterosexual males. Moreover, as the phenomenon of sexual robots will affect the whole of society, the control groups enabled us to gather first data on the initial reactions to sexual robots of a broader range of people (e.g., see Szczuka and Krämer, 2018, for a study on jealousy-related reactions of women).

#### Visual Attention Toward the Chest and Pelvic Region of Women and Robots Depending on Participants’ Gender and/or Sexuality

In terms of evolutionary psychological processes of perception, the chest and the pelvic region have been shown to be important in terms of providing information that is useful to estimate a person’s mate value (see section “Torso: Waist-to-Hip Ratio and Breasts” for details). The chest not only provides valuable information in terms of a woman’s age and potential sexual maturity, but also has a special meaning for the female body, as it is one of the most visible signs of the female gender and is consequently frequently used to display femininity and sexuality in the media. The pelvic region is of special visual importance as it is part of the waist-to-hip ratio and therefore serves as important guidance in terms of female health and a woman’s reproductive value. Based on the assumption that men have internalized the meaning of these body areas and are therefore drawn to look at them, we argue that heterosexual men will spend more time looking at these areas compared to heterosexual female and homosexual male participants. Moreover, we further argue that for this reason, heterosexual males will spend more time looking at the chest and the pelvic region of females than at the robotic replications of these body areas. Besides processes of visual attention driven by mate selection, it is conceivable that familiarity will positively affect the gaze behavior toward the female stimuli, because with humans, people can apply an internalized gaze pattern, instead of being confronted with something new that potentially confuses the way people visually gather information (see Hypothesis 3 for further details). Based on this, we derived the following hypotheses:

*H1: Compared to homosexual men and heterosexual women, heterosexual men will spend more time looking at the chest of women compared to human-like gynoid and machine-like gynoid robots.*
*H2: Compared to homosexual men and heterosexual women, heterosexual men will spend more time looking at the pelvic region of women compared to human-like gynoid and machine-like gynoid robots.*

Regarding the difference between the two robotic versions, it is conceivable that the visual similarity between the human-like robots and actual women will lead to a more similar gaze behavior compared to the machine-like robots. However, it is also conceivable that the physique of the robots, which also includes secondary sexual characteristics, may contribute to an evolutionary psychologically driven initial reaction of visual attention. This would be in line with Szczuka and Krämer (2017), who found no differences between women and female-looking robots (with salient mechanical body parts) in terms of the associative strength of the concept of attractiveness, which the authors argued was explained by visual cues. However, it might also be the case that salient mechanical body parts (e.g., a mechanical body in contrast to silicone) will break the visual illusion of a female entity and lead to a different form of visual attention compared to the empirically investigated gaze behavior of males toward women. Based on these conflicting arguments, we derived the following research questions:

*RQ1: Is there a difference in the time participants spend looking at the chest of the human-like and the machine-like robots?*
*RQ2: Is there a difference in the time participants spend looking at the pelvic region of the human-like and the machine-like robots?*

#### Visual Attention Toward the Head of Women and Robots

While the chest and the pelvic region of a woman provides information which is of particular interest for heterosexual men in terms of mating, the head provides information about the emotional and motivational state, which is of interest for all individuals in order to anticipate whether a person has positive or negative intentions (Ohman et al., 2001). However, while there may not be a strong difference between participants in terms of the gaze duration toward the head, it is conceivable that there will be differences based on the human-likeness of the different stimuli. As the face provides a large amount of valuable information about a person’s state (e.g., health, age, reproductive value, emotions, motivations), it may be that the head of a human gains more visual attention compared to the head of a human-like or a machine-like robot, as the latter two are not capable of providing authentic information (e.g., in terms of information or emotion). On the contrary, one might argue that the face of a human-like robot in particular provides numerous detailed replications of facial characteristics (e.g., eyebrows made of hair or paint that resembles make-up), which may also gain visual attention due to its resemblance to humans and interest in terms of the question how well human facial features can be replicated. This is in line with the fact that the face is one of the most challenging areas to construct in humanoid robots (Bar-Cohen and Hanson, 2009). Based on these elaborations, the following research question was asked:

*RQ3: Is there a difference in the time people spend looking at the head of humans, human-like robots and machine-like robots?*

#### Confusion or Deeper Exploration of the Robots Compared to Women

Furthermore, we aimed to investigate whether observers would be more confused and/or whether they would visually explore the stimuli more deeply when looking at human-like and machine-like female-looking robots compared to at women. Since robots are not yet a part of most persons’ day-to-day lives, we hypothesized that the participants could be confused by the stimuli, as they are unconsciously aware that the ways in which people gather visual information from women do not apply to robots. Although it might be argued that the human-like robots are visually similar to women, any potential uncertainty was reduced in the present study, as we used a statement clarifying whether the participant was looking at another human being or at a robot. It may be the case that such clarification contributes to confusion, insofar as people do not know which visual areas are of importance regarding robots, in contrast to the internalized gaze behavior toward women. Moreover, it may be speculated that people are interested in visually investigating the new technology by switching back and forth between different parts of the body in order to gain as much visual information as possible. The visual process of looking at one particular area of a stimulus and subsequently returning to it later on was found to quantify not only confusion (Salminen et al., 2018) but also the process of visual exploration (Kiefer et al., 2014). Based on these elaborations, the following hypothesis was formulated:

*H3: There will be a deeper visual exploration in terms of switching back and forth between different body parts when individuals [heterosexual men, homosexual men, and heterosexual women] are looking at human-like and machine-like gynoid robots compared to at women.*

#### Influence of the Evaluation of the Attractiveness of Stimuli, Personality Traits, and/or Negative Attitude Toward the Concept of Robots on the Gaze Behavior Toward Robots

Additionally, we were interested in the question of whether the visual attention of the heterosexual males toward the head, chest, and pelvic region of the robots (human-like and machine-like) could be predicted by evaluations of the attractiveness of the robots and/or a negative attitude toward the concept of robots in general. Szczuka and Krämer (2017) found that attractiveness ratings of sexualized robots were partially predicted by a person’s general negative attitude toward robots. We therefore aimed to investigate whether negative evaluations of the robots’ attractiveness and/or participants’ general negative attitude toward robots would lead to a lack of visual attention toward areas which are usually important for gaining an impression of an interaction partner (head, chest, and pelvic area). Furthermore, we were interested in the influence of anthropomorphism, meaning people’s tendency to ascribe human characteristics to objects. In this respect, we assumed that the participants would look longer at the head, chest, and pelvic area of the robots if they generally anthropomorphize objects and therefore make them more human-like. However, as argued above, we also wished to investigate whether deep-rooted, evolutionary psychological processes of mate selection drive the visual attention toward women and robotic representations of females. It is therefore also conceivable that the visual attention toward a mechanical replication of humans cannot be predicted by evaluations of robots, which, moreover, are explicit and therefore biased by potential effects of social desirability. As it is unknown whether visual attention is influenced by personal characteristics or the evaluation of the robots, the following research questions were posed:

*RQ4a: Does a negative attitude toward robots, a tendency to anthropomorphize, and the attractiveness ratings of the human-like and machine-like gynoid robots explain variance in the time heterosexual male participants spend looking at the chest of the human-like and machine-like gynoid robots?*
*RQ4b: Does a negative attitude toward robots, a tendency to anthropomorphize, and the attractiveness ratings of the human-like and machine-like gynoid robots explain variance in the time heterosexual male participants spend looking at the pelvic area of the human-like and machine-like gynoid robots?*
*RQ4c: Does a negative attitude toward robots, a tendency to anthropomorphize, and the attractiveness ratings of the human-like and machine-like gynoid robots explain variance in the time heterosexual male participants spend looking at the head of the human-like and machine-like gynoid robots?*

## Materials and Methods

### Participants

To investigate how heterosexual men look at gynoid robots in contrast to looking at women, and also to compare the results to groups that have different viewing patterns based on their sexuality and gender, 17 heterosexual men, 12 homosexual men, and 20 heterosexual women took part in the study. The participants’ age ranged from 18 to 34 (*M* = 22.96, *SD* = 4.112). Thirty-three of the subjects were in a relationship, while 12 were single. To avoid hormonal influences on the attention to sexual stimuli for women as reported by Rupp and Wallen (2007), we only recruited female participants who were taking oral contraceptives. All participants were recruited at a large German university or in Facebook groups related to this university. All participants had normal or corrected-to-normal vision (e.g., wearing soft contact lenses). The calibration validation accuracy value (maximum 0.5° on either the x or y axes; Holmqvist et al., 2011) and the tracking ratio (of at least 75%) served as standards for the quality of the data and led to the exclusion of four datasets. Therefore, 45 datasets were used in the present analyses (15 heterosexual men, 12 homosexual men, and 18 heterosexual women). This is in line with the results of the power analysis which was used to compute the required sample size *a priori*. The results showed that a total of 40 participants were needed to a have 95% likelihood of detecting a moderate effect (*f* (V) = 0.50). The effect size was chosen based on previous eye tracking studies investigating the gaze behavior toward different body parts of humans such as Bolmont et al. (2014) who found moderate effects for the duration of fixations on different body parts.

### Measures

This section contains information on the standardized questionnaires used as well as an explanation of the eye-tracking system and the corresponding metrics. Please note that the experiment was carried out in German.

#### Sexual Orientation

While we explicitly requited for heterosexual female participants only, we asked for both, homosexual and heterosexual in the advertisement for the study. To ascertain the sexual orientation of the male subjects, a five-point Likert scale modification of the Kinsey Scale was used (Kinsey et al., 1948). The single item asks for participants’ sexual orientation, with answers ranging from 1 = *“homosexual,”* through 3 = *“bisexual,”* to 5 = *“heterosexual.”*

#### Tendency to Anthropomorphize Technological Objects

A self-developed scale was used to measure the tendency to treat everyday objects, such as smartphones, computers or cars, as if they were humans. The seven items and statements were partly inspired by a work of Neave et al. (2015) who also investigated influences of anthropomorphic tendencies. The items can be found in the Table 1. The participants were asked to rate their agreement with the questions and statements on a five-point Likert scale ranging from 1 = *“strongly disagree”* to 5 = *“strongly agree.”* The internal consistency (Cronbach’s alpha) was α = 0.754.

**Table 1:** Items used to measure the tendency to anthropomorphize technological objects.

Item
(1) I have experienced that some of my electronic devices (e.g., smartphone or computer) refused to cooperate.
(2) I think that my computer/printer would function properly if it would be needed.
(3) I think that my computer is slow on purpose after I insulted it.
(4) One of the reasons why I once bought a new car, or an electronical device was because I instantly perceived its friendly personality.
(5) I ask myself whether my car or my computer does appreciate if I clean it.
(6) Do you tend to show thankfulness toward technological devices (e.g., smartphone or computers) or your car if it provides service in difficult situations?
(7) I find it odd to attribute human characteristics to technological devices. (reversed)

#### Negative Attitude Toward Robots (NARS)

The NARS scale by Nomura et al. (2006) measures negative attitudes toward robots with regard to social/future implications, emotional attitudes, and action interactions. The scale consists of 14 items, such as *“I feel that if I depend on robots too much, something bad might happen,”* which participants rated on a five-point Likert scale ranging from 1 = *“strongly disagree”* to 5 = *“strongly agree.”* The Cronbach’s Alpha was α = 0.826.

#### Eye Tracking

The SMI RED 500 remote eye-tracker was used to measure the gaze behavior at a 250 Hz sampling rate. The participants had an approximate distance of 700 mm to the 22” stand-alone monitor, which displayed the stimulus material with a resolution of 1920 × 1200 pixels. The “SMI experiment Center” was used to set up the experiment. The software provided the necessary functionalities for the procedure (see section “Procedure” for details on the procedure) and the possibility to define the relevant areas of interest (AOI) (see section “Stimulus Material” for details on the stimulus material). To analyze the data (see Supplementary Data Sheet 1 for details), we used the software BeGaze and SPSS 22. Here, the dwell time served as most important metric, which is the total amount of time spent within one AOI. It is composed of both the fixation duration(s) and the time spent during saccades.

#### Evaluation of the Relevant Stimuli

After the eye-tracking task, the participants were again confronted with the relevant stimuli and asked to rate the pictures with regard to their physical appearance. The single item stating, *“I find the appearance of the [woman][robot] to be appealing”* was rated on a five-point Likert scale ranging from 1 = *“strongly disagree”* to 5 = *“strongly agree.”*

### Stimulus Material

**Figure 1:**
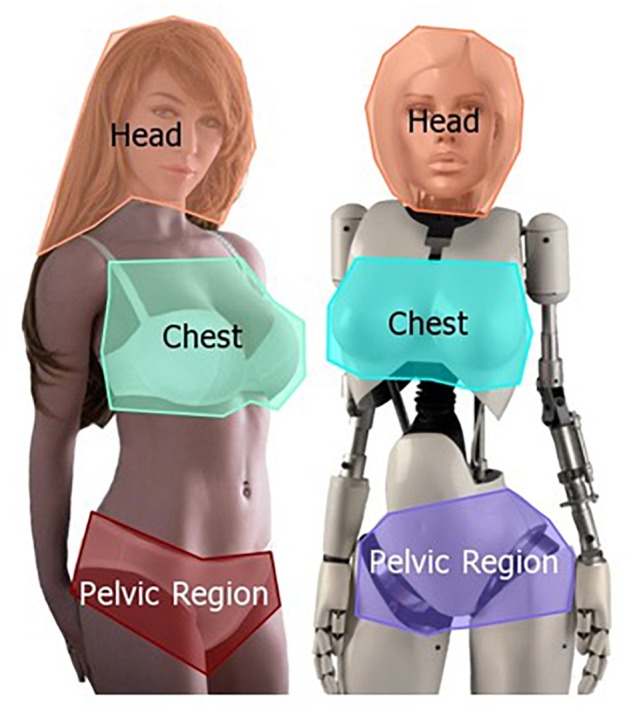
Examples of the human-like **(left)** and the machine-like **(right)** robotic stimuli. The author of the work holds the permission to use the pictures [copyright holder left picture: Sam Do (WM Dolls), copyright holder right picture: Jessica Szczuka]. Please note that due to missing consent, this figure does not show an example for the human stimuli.

To prevent the participants’ gaze from being influenced by repeated presentation of the secondary sexual characteristics of the women and gynoid robots or by the actual purpose of the study, the stimulus material consisted of 21 pictures. Six of the pictures were of relevance, showing the women and gynoid robots (human-like and machine-like), and the remaining 15 were irrelevant pictures showing male and female adults in basic clothing, as well as toy robots (e.g., Cozmo by Anki) and robotic animals (e.g., Erle-Spider by Erle Robotics). Two of the relevant pictures showed women in underwear, two displayed human-like gynoid robots in underwear, and two showed machine-like gynoid robots without underwear. The machine-like gynoid robots had no underwear because the robots on the pictures were made of white plastic and metal, and did not show any specific detail of the secondary sexual characteristics such as the breast papilla. This is in contrast to the human-like gynoid robots, which have silicone skin and therefore show anatomically correct details. It was important to include both kinds of gynoid robots as we wished to test whether the machine-likeness plays an important role for gaze behavior or whether basic visual cues (such as the shape) trigger the same viewing behavior as that when looking at a woman. All pictures shown, but especially the six essential stimuli showing women and female-looking robots, were chosen to be as comparable as possible. We paid attention to the background, the image section, the expression (e.g., smiling without showing teeth), basic underwear and the displayed waist-to-hip ratio. Additionally, the three most important AOIs head, chest and pelvic region were controlled with regard to their size in each stimulus group. The AOIs were chosen because these body regions have been found to provide important information about the fitness of women. Figure 1 shows an example for each robotic stimulus category and its AOIs.

### Procedure

The study consisted of three parts. First, the participants were informed about the procedure of the study and the data that was going to be assessed. In line with this the participants were informed about their rights (which are based on the ethical principles of psychologists of the American psychological association^1^). After signing informed consent, the participants were asked to answer a set of items assessing sociodemographic information and a questionnaire that measured the individual tendency to anthropomorphize everyday objects (see section “Measures” for details on the measurements). Subsequently, they were asked to watch a video clip of almost 90 s, which presented state-of-the-art robots such as Sophia by Hanson Robotics and HRP-4C (Miim) by the National Institute of Advanced Industrial Science and Technology (AIST). The video was used to create an understanding of how humanoid robots can nowadays look (e.g., having plastic or silicone skin and therefore having a more or less human-like appearance) and what their abilities are (e.g., expression non-verbal behavior, walking). This was important, as people tend to gather their information about and expectations toward robots from science fiction movies (Weiss et al., 2011). After the video, the second phase of the procedure took place as the experimenter started the eye-tracking task. In the first step, the ocular dominance was determined using the Miles ABC Test (Miles, 1929). Next, the participants were seated in front of the SMI RED 500 remote eye-tracker, which was used to record the gaze behavior (see section “Eye Tracking” for more details on the eye-tracking system). Participants were aware that they would see pictures of robots and humans but did not know the actual purpose of the study. As the study had a mixed factorial design, all participants saw the same 21 pictures, of which six were relevant (see section “Stimulus Material” for details on the stimulus material). They were instructed to look at the pictures of the robots and humans as they would normally do if, for instance, they saw such pictures in a magazine. After successful calibration and validation, the eye-tracking task began. The experiment was implemented in SMI Experiment Center, which has a function allowing it to automatically start the next trial whenever a picture has been looked at for long enough. In the present study, the participants had to look at every picture for 8000 ms before the next trial began automatically. This enabled the experimenter to leave the room, thus allowing the participants to look at the pictures without any experimenter bias. Before each picture was presented, a fixation cross appeared in the middle of the screen which participants needed to look at for 1500 ms, followed by a statement clarifying the nature of the upcoming stimulus (either *“In the following, you will see a human being”* or *“In the following, you will see a robot”*) for 2500 ms. This was important not only as the human-like gynoid robots look very human-like, but also because we wanted to trigger the mental schema of either humans or robots, even though some robots had a very human-like appearance. Afterward, one of the 21 pictures was shown for 8000 ms in a random position of the screen to avoid anticipatory saccades. This was followed by a blank screen presented for 1000 ms. This sequence was repeated until the participant had seen every picture. In the final part of the study, the subjects were asked to evaluate the stimulus material, before receiving a detailed debriefing. Please note that in order to meet the standards of ethical acceptability of psychological research, the study was approved by the ethics committee of the division of computer science and applied cognitive sciences of the University of Duisburg-Essen, Germany.

## Results

The study aimed at investigating whether evolutionary principles can explain how heterosexual men look at different body regions of female-looking robots in comparison to looking at women and whether the human-likeness of the robot makes a difference. Based on evolutionary psychological theories, the head, the chest and the pelvic region contain important visual information regarding the women’s potential mate value. To investigate differences in the gaze behavior based on gender and/or sexuality, the analyses not only focus on the gaze behavior of heterosexual men when viewing women in contrast to sexualized robots, but also include the two control groups (heterosexual women and homosexual men). The calculations are the same for the three main AOIs (head, chest, and pelvic region). Due to the mixed method study design, we first calculated 3x3 repeated measures ANOVAs with the stimulus groups (women, human-like gynoid robots and machine-like gynoid robots) as within-subject factor and the gender/sexuality of the participants (heterosexual men, homosexual men, and heterosexual women) as between-subject factor. To better understand the gaze pattern of the heterosexual men, we subsequently computed additional repeated measures ANOVAs for the data of the heterosexual men only. In the final step, we tested whether the personal characteristics negative attitude toward robots and the tendency to anthropomorphize, combined with the evaluation of the stimuli regarding their physical attractiveness, were predictors of the amount of time heterosexual males spend looking at the different AOIs. Moreover, we performed some additional calculations addressing deviations in the orientation when looking at the gynoid robots compared to at women, i.e., revisiting different areas of interest. Please note that we collapsed the data of the two pictures of each category (human, machine-like gynoid, human-like gynoid) into one common mean value in order to guarantee higher generalizability of the results. Table 2 provides a descriptive overview of how long the participants looked at the different AOIs of each stimulus group. The numbers represent the mean dwell times in milliseconds. Afterward, the analyses of the hypotheses will be explained in detail. Please note that we will provide a table at the end of the section summarizing the hypotheses and research questions in relation to the findings (see Table 3).

**Table 2:** Dwell times in milliseconds of all AOIs separated by stimulus and participant groups.

AOI	Stimulus groups	Participant groups					
		Heterosexual men		Homosexual men		Heterosexual women	
		*M*	*SD*	*M*	*SD*	*M*	*SD*
Head	Female stimuli	4035.68	1700.64	4558.01	1339.81	4223.79	1615.12
	Human-like robotic stimuli	3214.65	2096.91	4544.63	1571.25	3672.61	1532.59
	Machine-like robotic stimuli	3255.82	987.95	3572.96	1065.23	3582.71	1281.06
Chest	Female stimuli	1278.72	853.03	863.25	525.32	963.90	560.12
	Human-like robotic stimuli	1074.54	553.46	961.61	603.04	1015.58	488.87
	Machine-like robotic stimuli	780.08	544.16	906.42	485.25	763.82	488.11
Pelvic region	Female stimuli	501.46	301.13	346.33	232.08	380.44	301.36
	Human-like robotic stimuli	635.45	418.24	232.30	205.08	362.54	203.82
	Machine-like robotic stimuli	693.97	380.57	518.50	220.85	640.27	318.19
Body elsewhere (arms, legs, abdomen)	Female stimuli	863.77	473.76	878.71	546.14	1170.78	936.03
	Human-like robotic stimuli	1231.32	653.67	878.65	579.69	1248.78	621.22
	Machine-like robotic stimuli	1269.16	696.69	990.57	379.82	1003.98	449.81
Background	Female stimuli	626.74	593.65	741.29	313.77	554.62	341.22
	Human-like robotic stimuli	919.63	628.83	750.45	450.53	833.82	572.98
	Machine-like robotic stimuli	846.73	227.17	1289.55	948.09	1185.71	597.71

**Table 3:** Summary of the hypotheses/research questions and the findings.

Emphasis and hypothesis/research question	Findings
**Chest region**	• No main effect gender/sexuality
	• Viewing times all participants:
*H1:* Compared to homosexual men and heterosexual women, heterosexual men will spend more time looking at the chest of women compared to human-like gynoid and machine-like gynoid robots.	Female chest >
*RQ1:* Is there a difference in the time participants spend looking at the chest of the human-like and the machine-like robots?	Human-like robotic chest > Machine-like robotic chest
	• Viewing times heterosexual males only:
	Female chest >
	Human-like robotic chest = Machine-like robotic chest
**Pelvic region**	• Main Effect gender/sexuality: heterosexual males = more viewing time at the pelvic area of the human-like robot than homosexual males and heterosexual women
	• Viewing times all participants:
*H2:* Compared to homosexual men and heterosexual women, heterosexual men will spend more time looking at the pelvic region of women compared to human-like gynoid and machine-like gynoid robots.	Human pelvic region <
*RQ2:* Is there a difference in the time participants spend looking at the pelvic region of the human-like and the machine-like robots?	Human-like robotic pelvic region > Machine-like robotic pelvic region
	• Viewing times heterosexual males only:
	Human pelvic region <
	Human-like robotic pelvic region = Machine-like robotic pelvic region
**Head**	• Viewing times all participants:
*RQ3:* Is there a difference in the time people spend looking at the head of humans, human-like robots and machine-like robots?	Female head >
	Human-like robotic head > Machine-like robotic head
	• Viewing times heterosexual males only:
	Female head >
	Human-like robotic head > Machine-like robotic head
**Need for visual exploration**	• Revisits:
*H3:* There will be a deeper visual exploration in terms of switching back and forth between different body parts when individuals [heterosexual men, homosexual men, and heterosexual women] are looking at human-like and machine-like gynoid robots compared to at women.	Female Stimuli <
	Human-like robots = machine-like robots
**Influence of evaluations, personality traits and attitudes toward robots**	• Viewing times toward chest of human-like robot = Regression model not significant
*RQ4a:* Does a negative attitude toward robots, a tendency to anthropomorphize, and the attractiveness ratings of the human-like and machine-like gynoid robots explain variance in the time heterosexual male participants spend looking at the chest of the human-like and machine-like gynoid robots?	
	• Viewing times toward chest of machine-like robot = Regression model not significant
*RQ4b:* Does a negative attitude toward robots, a tendency to anthropomorphize, and the attractiveness ratings of the human-like and machine-like gynoid robots explain variance in the time heterosexual male participants spend looking at the pelvic area of the human-like and machine-like gynoid robots?	• Viewing times toward pelvic area of human-like robot = Regression model not significant
	• Viewing times toward pelvic area of machine-like robot = Regression model not significant
*RQ4c:* Does a negative attitude toward robots, a tendency to anthropomorphize, and the attractiveness ratings of the human-like and machine-like gynoid robots explain variance in the time heterosexual male participants spend looking at the head of the human-like and machine-like gynoid robots?	• Viewing times toward head of human-like robot = Regression model not significant
	• Viewing times toward head of machine-like robot = Regression model not significant

### Visual Attention Toward the Female and Robotic Chest (H1 and RQ1)

**Figure 2:**
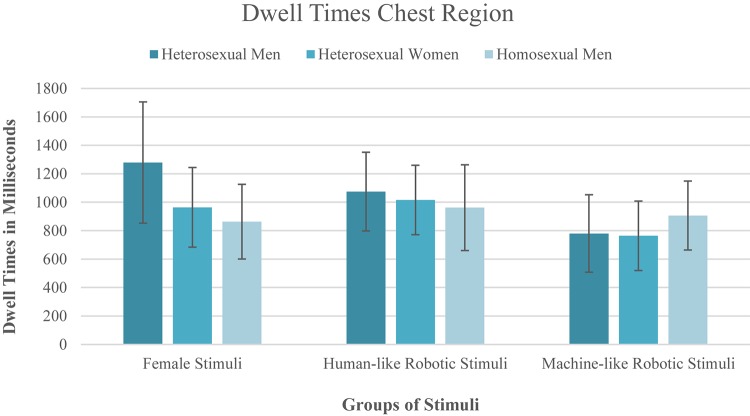
Illustration of the dwell times on the different chest regions.

A 3x3 mixed design repeated measures ANOVA was conducted to test potential differences in the time spent looking at the chest region of women, android robots and humanoid robots in relation to the participants’ gender and their sexual orientation (H1a). The dwell time on the chest region of the three different stimulus groups (women vs. human-like gynoid robots vs. machine-like gynoid robots) was used as within-subject factor and gender/sexuality of the participants (heterosexual women, heterosexual men, and homosexual men) as between-subject factor. Mauchly’s test revealed that the assumption of sphericity was met (χ^2^(2) = 5.78, *p* = 0.056) and Levene’s test showed that the assumption of homogeneity of variance was met for all three dependent variables. There was a marginally significant difference between the stimulus groups (*f* (2,84) = 3.04, *p* = 0.053, $\eta_{p}^{2}$ = 0.068). The Helmert contrasts showed no significant difference in the time the participants spent looking at the chest region of the woman compared to the chests of the two robotic versions (*f* (1,42) = 2.12, *p* = 0.152, $\eta_{p}^{2}$ = 0.048). However, a difference did emerge with respect to the gynoid robots: the participants spent significantly more time looking at the chest of the human-like gynoid robots than at the chest of the machine-like gynoid robots (*f* (1,42) = 3.80, *p* = 0.058, $\eta_{p}^{2}$ = 0.083), although this was only significant at the 10% level. The main effect of participants’ gender/sexual orientation was not significant (*f* (2,42) = 0.46, *p* = 0.636, $\eta_{p}^{2}$ = 0.021). Moreover, there was no significant interaction effect of stimulus group × participants’ gender/sexuality (*f* (4,84) = 1.26, *p* = 0.294, $\eta_{p}^{2}$ = 0.056). Figure 2 illustrates the differences between the participants’ gender/sexuality and the stimulus groups.

As we were particularly interested in the question of whether different types of gynoid robots trigger the same viewing pattern in men as the viewing pattern triggered by women, we ran an additional repeated measures ANOVA with the data of the heterosexual men only. The assumption of sphericity was met (χ^2^(2) = 4.953, *p* = 0.084). In contrast to the results for all participants, we found a significant difference between the time the heterosexual men spent looking at the women’s and robots’ chest regions (*f* (2,28) = 3.69, *p* = 0.038, $\eta_{p}^{2}$ = 0.208). The Helmert contrast showed that heterosexual men spent significantly more time looking at the chest of the women compared to the chest region of the two types of robots (*f* (1,14) = 4.81, *p* = 0.046, $\eta_{p}^{2}$ = 0.256). No significant difference emerged between the human-like and machine like robots in terms of dwell time on the chest region (*f* (1,14) = 2.550, *p* = 0.133, $\eta_{p}^{2}$ = 0.154).

All in all, the results partly supported the first hypothesis. While the data of the heterosexual males only showed that the males did indeed look at the chest of the female stimuli for longer than at the chests of the robots, there was no main effect of the participants’ gender. Regarding Research Question 1, the results showed no difference in the time the heterosexual males spent looking at the human-like and machine-like robotic chest. However, if the data from all participants were included, there was a difference in the visual attention toward the two robotic chest regions.

### Visual Attention Toward the Female and Robotic Pelvic Region (H2 and RQ2)

**Figure 3:**
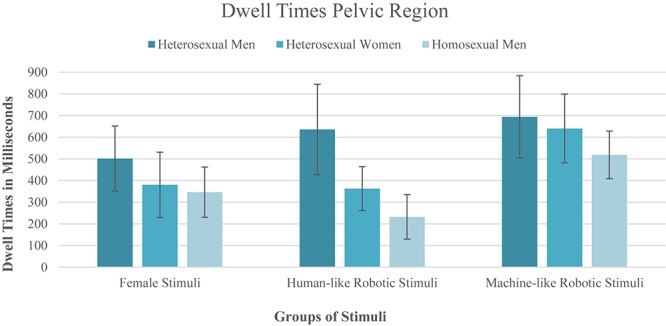
Illustration of the dwell times on the different pelvic regions.

To investigate whether dwell times on different pelvic regions differed according to participants’ gender/sexual orientation, a 3x3 mixed design repeated measures ANOVA was conducted, with the dwell times on the pelvic regions (woman vs. human-like gynoid robot vs. machine-like gynoid robot) as within-subject factor and participants’ gender/sexuality (heterosexual women, heterosexual men and homosexual men) as between-subjects factor. The assumption of sphericity was met according to Mauchly’s test (χ^2^(2) = 1.59, *p* = 0.451), but the assumption of homogeneity of variance was violated for one of the three within-subject variables (dwell time for the pelvic region of the android robot, *F* = 4.45, *p* = 0.018). According to Field (2013), this is acceptable, as the group sizes are nearly equal. The results revealed a significant main effect of participants’ gender/sexuality (*f* (2,42) = 4.15, *p* = 0.023, $\eta_{p}^{2}$ = 0.165). The Bonferroni *post hoc* test of an additional one-factor ANOVA showed no significant difference in the amount of time the heterosexual women, the heterosexual men and the homosexual men spent looking at the pelvic region of the female stimuli and the machine-like gynoid robots. The amount of time participants spent looking at the pelvic region of the human-like gynoid robots did show a significant difference with respect to gender/sexual orientation: heterosexual men looked significantly longer at the pelvic region of the human-like robots than did heterosexual women (*p* = 0.033) and homosexual men (*p* = 0.003). In line with this, the 3x3 mixed design repeated measures ANOVA yielded a main effect of stimulus group (time the participants spent looking at the human pelvic region, at the human-like gynoid robot’s replication of the pelvic region and at the machine-like gynoid robot’s replication of the pelvic region) (*f* (2,84) = 10.61, *p* < 0.001, $\eta_{p}^{2}$ = 0.202). Planned Helmert contrasts revealed that the dwell times directed toward the pelvic regions of the women were significantly shorter than the dwell times directed toward the replicated pelvic regions of the two types of robot (*f* (1,42) = 6.10, *p* = 0.018, $\eta_{p}^{2}$ = 0.127). Moreover, planned contrasts also showed that the participants looked significantly longer at the replicated pelvic region of the machine-like robot compared to that of the human-like gynoid robot (*f* (1,42) = 14.14, *p* = 0.001, $\eta_{p}^{2}$ = 0.252). However, no gender × stimulus group interaction was found (*f* (4,84) = 1.390, *p* = 0.246, $\eta_{p}^{2}$ = 0.062). Figure 3 shows the differences of the dwell times toward the pelvic regions between the different stimulus groups in terms of participants’ gender/sexuality.

As explained above, we were particularly interested in the viewing behavior of the heterosexual men. To test whether the effects found overall could be replicated for the heterosexual men only, we conducted another repeated measures ANOVA. The assumption of sphericity was met (χ^2^(2) = 1.98, *p* = 0.373). Although the main effect of stimulus group was non-significant (*f* (2,28) = 1.76, *p* = 0.190, $\eta_{p}^{2}$ = 0.112), the contrast revealed differences in the time participants spent looking at the different stimuli. Heterosexual men also spent significantly less time looking at the pelvic region of the women compared to the two gynoid robots (*f* (1,14) = 4.45, *p* = 0.053, $\eta_{p}^{2}$ = 0.241). However, in contrast to the analysis of all participants, there was no significant difference in the time heterosexual men spent looking at the pelvic regions of the human-like and the machine-like gynoid robots (*f* (1,14) = 0.24, *p* = 0.630, $\eta_{p}^{2}$ = 0.017).

Taken together, Hypothesis 2 could not be confirmed. Contrary to our assumption, the results revealed that participants did not show higher visual attention toward authentic information of the human stimuli compared to the robotic stimuli. This was the case both for heterosexual male participants only and for all participants combined. With regard to Research Question 2, there was a difference in the time spent looking at the two robotic pelvic areas, with longer dwell times on the machine-like robots. However, this difference was not confirmed for the heterosexual males only.

### Visual Attention Toward the Female and Robotic Head (RQ3)

**Figure 4:**
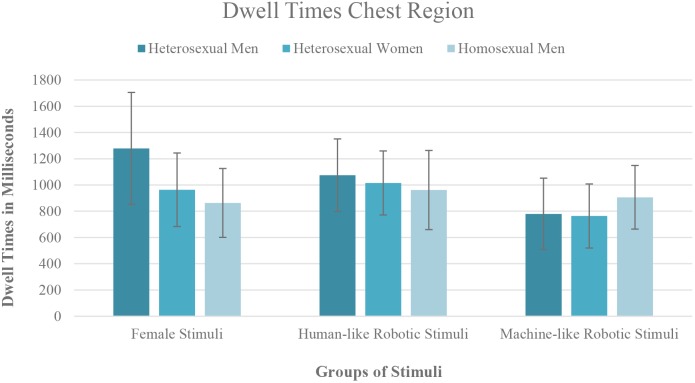
Illustration of the dwell times on the different heads.

Based on the third research question, which asked whether there is a difference in the time people spend looking at the head of a woman compared to the head of human- and machine-like robots, a repeated measures ANOVA was conducted with the viewing times directed toward the heads of the women, the human-like gynoid robots and the machine-like gynoid robots as within-subject factor. As the sphericity assumption was violated (χ^2^(2) = 8.56, *p* = 0.014) and the Greenhouse–Geisser ε was above 0.75, the Huynh-Feldt correction was used (ε = 0.87) (Field, 2013). The results showed a significant main effect for the three different types of head (*f* (1,83,76.85) = 7.140, *p* = 0.002, $\eta_{p}^{2}$ = 0.145). The additionally computed Helmert contrasts showed that the participants spent significantly more time looking at the heads of the women compared to the heads of the two types of robot (*f* (1,42) = 16.74, *p* < 0.001, $\eta_{p}^{2}$ = 0.285). Additionally, no significant effect was found regarding the time the participants spent looking at the heads of the two robot versions (*f* (1,44) = 1.96, *p* = 0.169, $\eta_{p}^{2}$ = 0.045). No significant main effect was found for the participants’ gender/sexuality (*f* (2,42) = 1.08, *p* = 0.350, $\eta_{p}^{2}$ = 0.049), and the interaction between stimulus group and participants’ gender/sexuality was also non-significant (*f* (3.66,76.85) = 1.11, *p* = 0.356, $\eta_{p}^{2}$ = 0.050). Figure 4 illustrates the differences in the gaze behavior between the stimulus groups depending on participants’ gender/sexuality.

To gain a better understanding of how the heterosexual men looked at the human and non-human heads, an additional repeated measures ANOVA was conducted with the data of the heterosexual men only. As the assumption of sphericity was not met (χ^2^(2) = 6.219, *p* = 0.045), Greenhouse–Geisser correction was applied (ε = 0.73). Although the main effect of stimulus group was non-significant (*f* (1.45,20.29) = 2.38, *p* = 0.130, $\eta_{p}^{2}$ = 0.145), the Helmert contrasts showed the same pattern as in the analyses with all participants as reported above; the heterosexual men spent significantly more time looking at the head of the women compared to the two robotic heads (*f* (1,14) = 9.08, *p* = 0.009, $\eta_{p}^{2}$ = 0.393). However, there was no significant difference in the time the heterosexual males spent looking at the human-like and machine-like robots (*f* (1,14) = 0.01, *p* = 0.938, $\eta_{p}^{2}$ = 0.000).

Overall, the third research question can be answered by stating that all participants spent more time looking at the authentic information provided by the human heads than at both the human-like and machine-like robotic heads. No differences were found between the participant groups. However, the data of the heterosexual men only confirmed the higher visual attention toward the human heads in comparison to the two robotic heads, but did not reveal any difference in visual attention toward the human- and machine-like robots.

### Exploration of the Robots Compared to Women (H3)

To statistically examine whether the participants switched between the different AOIs more often when they were viewing gynoid robots, because they did not draw on the mental framework activated when looking at a woman (H3), we conducted a 3x3 mixed design repeated measures ANOVA. For this purpose, three sum scores were computed, including all revisits to all AOIs of one stimulus category (head, chest region, pelvic region, body elsewhere). A revisit is defined as returning the gaze to an area of interest which the person has already viewed. Each revisit sum score of the three stimulus categories (women, human-like gynoid robots, and machine-like gynoid robots) served as within-subject factor and participants’ gender/sexuality (heterosexual women, heterosexual men, and homosexual men) as between-subject factor. The assumptions of sphericity and homogeneity of variance were both met. Although there was no main effect of participants’ gender/sexual orientation (*f* (2,42) = 0.10, *p* = 908, $\eta_{p}^{2}$ = 0.005), the difference between the stimulus categories turned out to be significant (*f* (2,84) = 3.01, *p* = 0.055, $\eta_{p}^{2}$ = 0.067). The Helmert contrasts revealed that the participants revisited the different AOIs of the women significantly less compared to the two kinds of robot (*f* (1,42) = 8.66, *p* = 0.005, $\eta_{p}^{2}$ = 0.171), while there was no significant difference between the two robot types (*f* (1,42) = 0.03, *p* = 0.870, $\eta_{p}^{2}$ = 0.001). No gender × stimulus group interaction effect was found (*f* (4,84) = 0.872, *p* = 0.484, $\eta_{p}^{2}$ = 0.040). Taken together, Hypothesis 3 was confirmed.

### Influence of the Evaluation of the Attractiveness of the Stimuli, Personality Traits and/or Negative Attitude Toward the Concept of Robots on the Gaze Behavior Toward Robots (RQ4 a, b, c)

To test research question 4a, which asked whether the viewing times of the heterosexual male participants toward the chests of the robots (human-like and machine-like) could be predicted by the negative attitude toward robots, the individual tendency to anthropomorphize and/or the evaluation of the robots’ attractiveness (RQ4a), we conducted two linear regressions. Although the prediction model including the dwell times on the human-like robots was non-significant (*f* (3,11) = 1.610, *p* = 0.243, *R*^2^ = 0.305), the negative attitude toward robots turned out to be a marginally significant negative predictor of the time the heterosexual male participants spent looking at the chest of the human-like gynoid robot (β = -0.544, *p* = 0.085). However, none of the included variables was able to predict the time the heterosexual males looked at the machine-like robot’s chest (*f* (3,11) = 1.203, *p* = 0.354, *R*^2^ = 0.247).

Two linear regressions were computed to test whether the viewing times of the heterosexual men toward the pelvic regions of the two robotic versions could be predicted by their negative attitude toward robots and individual tendency to anthropomorphize, as well as by their evaluation of the robots regarding physical attractiveness (RQ4b). The regression model with the dwell time on the pelvic regions of the human-like gynoid robots as dependent variable failed to reach significance (*f* (3,11) = 1.051, *p* = 0.409, *R*^2^ = 0.223), and the model for machine-like gynoid robots was also non-significant (*f* (3,11) = 1.280, *p* = 0.330, *R*^2^ = 0.259).

To test whether personal characteristics such as negative attitude toward robots and individual tendency to anthropomorphize, combined with the evaluation of robots’ attractiveness, predict the time which heterosexual men spend looking at the head of the robots (RQ2c), two linear regressions were computed. The predictors were non-significant both for the human-like gynoid robots (*f* (3,11) = 1.03, *p* = 0.418, *R*^2^ = 0.22), and for the machine-like gynoid robots (*f* (3,11) = 1.86, *p* = 0.196, *R*^2^ = 0.34).

Taken together, none of the included variables could be identified as an overarching predictor of the heterosexual males’ dwell times on the replications of the chest, pelvic area and heads of both human-like and machine-like robots.

### Summary of the Hypotheses and Research Questions in Relation to the Findings

The following table (see Table 3 for details) summarizes the hypotheses ad research questions in relation to the findings. Just as within the results section, the order of the content will be based on the different body areas, followed by analyses that were independent of specific body regions of the stimuli.

## Discussion

### Visual Attention Toward Women in Comparison to Robots (H1, H2, RQ1, RQ2, H3)

In the following, we discuss the gaze behavior toward the chest, the pelvic region and the head of the female stimuli in comparison to both human-like and machine-like gynoid robots. Subsequently, we derive more general implications of the gaze behavior toward females in comparison to robots in terms both of theory and of the handling of sexualized robots.

The results of the present study regarding the visual attention toward the chest region of the human stimuli, the human-like and the machine-like robots, revealed differences in the gaze pattern based on gender and sexual orientation. As part of these differences, it became apparent that only heterosexual males spent significantly more time gathering information from the chest region of the female stimuli compared to the robotic versions. One likely explanation for this is that human breasts provide authentic visual information (which is important in order to efficiently estimate a person’s mate value); robots cannot provide such information and therefore do not require a deeper visual exploration. The importance of authenticity is underlined by the fact that differences emerged despite the strong resemblance of this region (in terms of shape and color). Contrary to this explanation, it may also be that the shorter viewing times toward the robotic chests could represent a lack of interest, or the lack of a perceptual strategy that is needed if one is confronted with a potential mate that is not human. Overall, this result provides first empirical evidence suggesting different perceptual processes of a potential mate based on the categorical assessment as human or non-human. Moreover, when considering the data of all participants, we found that all participants (including homosexual men and heterosexual women) spent marginally more time looking at the human-like breasts compared to those of the machine-like robots, while there was no difference in the time all participants spent looking at the chest region of the women compared to the female-like robots. Interestingly, the descriptive data revealed that the heterosexual women were particularly interested in gaining visual information from the chest region of the human like-robots. It is conceivable that the women were especially interested in the artificial implementation of the breasts, as they are a very important representation of femininity (Millsted and Frith, 2003). As breasts are secondary sexual characteristics that are frequently used to visually deliver messages connected to sex (e.g., in advertisements), it may be that the artificial human-like breasts helped women to estimate the robot’s qualities in terms of how sexual characteristics are implemented. This information could potentially also be used to compare the self to the replication as a form of a comparison of the species. Research has already demonstrated that females do compare themselves to female-looking robots (Szczuka and Krämer, 2018).

In terms of the pelvic area of the stimuli, we assumed that the heterosexual males would have a higher need for visual information from the pelvic area of the women, as this body area provides visual information on female sexual maturity and health (Singh, 1993; Buss, 1999). However, the results showed a completely different gaze behavior than assumed. The findings indicated that the robotic pelvic regions gained more visual attention compared to that of the women, regardless of participants’ gender and/or sexuality. Moreover, the analysis of the viewing times toward the robotic pelvic areas revealed that the machine-like robots gained more visual attention than the human-like ones. However, when reconsidering the pictures, it became apparent that the machine-like robots showed mechanical body parts connecting the torso and the legs (see section “Stimulus Material” for details on the stimulus material). Furthermore, as the machine-like robots were displayed with no skin, in contrast to the human-like robots, hinges and technical details were visible. It is therefore likely that the results regarding the pelvic area can first and foremost be interpreted as a sign that the salient mechanical body parts of the machine-like robots raised participants’ curiosity and the need for a deeper exploration of the technical details.

Regarding the influence of gender/sexuality, we found differences in the time participants spent looking at the human-like robotic pelvic regions insofar as heterosexual men looked significantly longer than did homosexual men and heterosexual women. It is possible that the heterosexual males were most interested in this area because they were the only participant group who would theoretically have intimate physical contact with the pelvic area during sexual interactions. It is therefore likely that the prolonged gaze times stemmed from curiosity and the need to explore the quality of the robot as a sexual interaction partner.

With regard to the head, we found that all individuals, regardless of their gender and sexuality, spent more time looking at human heads compared to robotic ones (human-like and machine-like). While one may have imagined that the details which are required to replicate a face would gain visual attention (especially with regard to the human-like robots, see Bar-Cohen and Hanson, 2009), it rather seems to be important what lies behind the facial characteristics and impressions. A human face provides valuable information not only about the person’s health or age (Buss, 1999; Stephen et al., 2009) but also about the person’s motivational and emotional state. The latter is especially important in order to efficiently estimate whether the individual is well-meaning or should be avoided (e.g., because he or she has an angry/aggressive expression; Ohman et al., 2001). Contrary to this, a robotic head is not capable of providing any authentic information that would help the user to evaluate whether the robot might harm a person. This is strongly linked to an alternative explanation, which is that humans have not yet developed a strategy on how to gain valuable information if confronted with a robot. In contrast to humans, who express their emotional state in terms of non-verbal behavior (Ekman et al., 1972; Darwin, 2000), a robot’s expression can be unrelated to its internal state. It is apparent that robots’ facial expressions are rather products of the implementations of movements and do not represent any internal emotional states. Based on this, it is questionable whether humans will find a strategy to efficiently gather information from a robot based on its appearance, or whether having access to the underlying computational processes will be the only solution to gain knowledge about a robot’s capabilities, consequently helping the user to evaluate whether he or she wants to have an interaction with it.

The analyzed gaze pattern also needs to be discussed in terms of the theoretical background upon which the study is based. The results of the present study did not support the hypothesis of equal gaze patterns toward machines and to humans, which we derived from media equation assumptions (Reeves and Nass, 1996). Even if humans have the tendency to behave socially in interactions with technology if they provide a sufficient number of social cues (Nass and Moon, 2000), they seem to have different strategies to gather important information from the technology compared to the processes of visual perception among humans. The lack of difference in the viewing times toward the face of the human-like and machine-like robots, moreover, underlines that the human-like appearance of something artificial does not automatically contribute to reactions that are similar to processes that are evoked if individuals are confronted with other humans. It is surprising that the human-like robot, which is equipped with facial details that would enable the imitation of facial expressions did not evoked more visual attention in comparison to the machine-like robots. Future research needs to investigate whether this might constitute first evidence that throughout sexualized interactions, humans unconsciously react differently toward robots than toward other humans, even though they might actively interact with them.

Regarding the avoidance-approach conflict (Lewin, 1935), the eye-tracking results of the present study give reasons to believe that machine-like cues, which strongly display the mechanical nature of the robot, do not have an influence on processes of avoidance, as we found no significant difference in the time the participants spent looking at the head and chest of the human-like and machine-like robots. However, the results also imply that robots do not create the same positive draw to body areas that are important in terms of mating (chest and head). Studies in which people have ongoing interactions with sexualized robots may help to achieve a deeper understanding of processes of avoidance and approach in terms of sexualized robots.

### Confusion or Deeper Exploration of the Robots Compared to Women (H3)

As already discussed in the context of the results, we assumed that the knowledge about the nature of the stimuli would activate deep-rooted processes of visual attention. We further argued that individuals would require a deeper exploration of the robotic stimuli in compared to the displayed human stimuli. The hypothesis was confirmed, as participants switched significantly less from one area of interest to another and back when confronted with human stimuli compared to non-human stimuli. Although one may have assumed that the number of revisits to the different areas of interest would have been smaller for the human-like robots than for the machine-like robots because the former have a stronger resemblance to humans, the results showed no significant difference. This is especially interesting as the human-like robots are very similar in appearance to humans but were marked as robots in the experiment in order to be identifiable as artificial. The present findings therefore underline that the concept “robot” is indeed categorized as something different than humans and is consequently accompanied by different processes of perception. Given that so far, only a minority of people have ever interacted with a robot (Bruckenberger et al., 2013) and that most people have probably never been confronted with a sexualized android robot, it seems plausible that people have a need to gather information on a technological device with which they are not (yet) familiar. As we scrutinized the participants’ initial reactions, it can be assumed that the non-human stimuli were understood as something other than human, but also that participants were interested in gaining visual information from different body parts of the robots, consequently representing a mixture of both confusion and the need for further exploration.

### Influence of the Evaluation of the Attractiveness of the Stimuli, Personality Traits and/or Negative Attitude Toward the Concept of Robots on the Gaze Behavior Toward Robots (RQ4 a, b, c)

To achieve a deeper understanding of potential influences on heterosexual males’ gaze behavior toward sexualized robots, the present study also aimed to investigate whether the explicit attractiveness ratings of the robots, the negative attitude toward robots and the tendency to anthropomorphize technology would explain the visual attention toward the body areas of the robots which are particularly relevant for mating among humans. The attractiveness ratings were included based on the idea that heterosexual male participants would spend more time looking at the body areas relevant for mating if they found the stimuli sexually attractive. The results of the present study yield no support for this hypothesis with regard to either the human-like or machine-like robots. However, it is conceivable that the explicit attractiveness ratings of the robots were influenced by social desirability. On the one hand, some heterosexual males might feel inclined to rate the robot less positively, as rating the robots as sexually attractive would violate societal and sexual norms. In line with this, Szczuka and Krämer (2017) revealed differences between explicit and implicit ratings of attractiveness of sexualized robots in comparison to woman. In their study, heterosexual males showed no differences between robots and women regarding the associative strength of the concept of attractiveness, but found significantly higher attractiveness ratings for women compared to human-like and machine-like ratings when participants were asked explicitly. On the other hand, it may also be the case that especially heterosexual males might have a bias to rate the robots as attractive because they overestimate their openness toward intimate interactions with something other than humans in order to underline their masculinity and the stereotypically connected importance of sexual interactions (Byers, 1996). Consequently, it is conceivable that the resulting discrepancy in the validity of the used explicit and implicit measures potentially had an effect on the relation of the variables.

Moreover, the analyses demonstrated that the gaze behavior toward the sexualized robots was not predicted by a negative attitude toward robots. Analyses yielded only a marginally significant effect regarding the chest of the human-like robots, indicating that there is a link between negative attitude toward robots and a subsequent lack of interest in exploring strongly sexualized body parts of human-like robots. This is in line with a study by Richards et al. (2017), who found that a negative attitude toward robots was a negative predictor of the explicitly evaluated likelihood of (hypothetically) participating in a sexual experience with a robot. However, this is only a faint possibility, as the negative attitude toward robots did not predict the visual attention toward the head or the pelvic area of the robots. In general, it cannot be excluded that this finding was again influenced by the lack of validity of explicit measurements.

Furthermore, we anticipated that the tendency to anthropomorphize technology would influence how men perceive robots in terms of human-likeness and would therefore predict the time participants spend looking at body parts of robots that have been shown to be important for mating among humans. The results did not support this assumption. It can be speculated that anthropomorphization, which is described as the tendency to treat objects like humans, is more important in social interactions than in initial perceptions. This is supported by the fact that the items used in the present study to measure anthropomorphization focused more strongly on behaviors and attitudes of people toward objects (e.g., “*I have experienced that some of my electronic devices (e.g., smartphone or computer) refused to cooperate*”).

### Limitations and Future Studies

The study is not without limitations. Although we applied a within-subjects design and conducted a power analysis to ensure that the sample was sufficiently large, a greater sample size might have positively influenced the generalizability and variability of the data.

It may be criticized that the artificial nature of the observation situation could have influenced how the individuals looked at the stimulus material. However, we tried to avoid such an influence by employing an experimental design in which the experimenter left the room and in which the participants themselves took control of the viewing task.

Moreover, a larger variance in the stimulus groups, and more specifically a higher number of displayed females, human-like robots and machine-like robots, might potentially have reduced the likelihood of effects that are based on features of specific pictures.

As the present study constitutes a first attempt to gain knowledge about deep-rooted reactions to sexualized robots, it raises further questions to be tackled in future research. One of the most important open questions is whether the fact that we explicitly informed participants about the nature of each picture may have influenced the gaze behavior toward the robots. As we did not find unambiguous significant differences in the gaze behavior toward human-like and machine-like robots, it appears that prior knowledge about the nature of the category led to different gaze behaviors, regardless of the human-like cues. Future research therefore needs to scrutinize the relative importance of categorical perceptions compared to deep-rooted reactions to human-like cues.

Future studies should consider more diverse samples with regard to different aspects. First with regard to the participants sexuality/gender. While the study included heterosexual women as a control group in order to investigate evolutionary psychological mechanisms of mate perception among heterosexual males, future studies could more explicitly target how heterosexual women perceive sexualized female replications. Future studies should also investigate how people of different sexualities perceive sexualized robots, by including robots representing different genders as research objects. Another aspect that is closely related to more diverse user groups that should be considered in future research is the need to investigate potential influences of the user’s ethnicity. Even though there is a lack of intercultural comparisons regarding perceptual processes of mate selection, there are first elaborations on why for instance Asian cultures might have a different understanding and evaluation in comparison to Western cultures (e.g., MacDorman et al., 2008). More research is needed in order to investigate whether this would also affect the way how human-like sexualized robots are perceived. Moreover, the samples of future studies should be more diverse with regard to age. As the participants in the present study can be considered to be rather young (18 to 34) more research is needed in order to investigate whether the results can be found in samples with a more diverse age structure. This should then also include a variation of the presented stimuli.

In general, sexualized robots are still in the early stages of development. As companies are currently working on convincing prototypes of sexualized robots that are capable not only of body movements but also of interactive conversations, future studies will have the opportunity to investigate perceptual, physiological or behavioral processes within a reciprocal human–robot interaction.

## Conclusion

Taken together, the present study provides empirical evidence that there is a difference in the way people initially perceive women in contrast to both human-like and machine-like gynoid robots. The data support the activation of deep-rooted mechanisms of visual attention depending on knowledge of whether the interaction partner is human or non-human. Heterosexual men spent more time gathering visual information regarding women’s face and chest compared to the robotic replications of these body areas, regardless of whether they were machine- or human-like. As these body areas have been shown to be important for efficiently estimating a woman’s mate value, our findings support the conclusion that heterosexual men do not transfer evolutionary psychological perceptual mechanisms of mate selection to robots. Moreover, it is noteworthy that this gaze pattern suggesting a preference for supposedly meaningful human stimuli was also observable among the homosexual men and heterosexual women, as all participants spent more time looking at the human faces compared to the robotic faces. It can be suggested that these differences in gaze behavior are based on the knowledge that robots are non-living entities, which are therefore not able to provide authentic visual information in terms of their biological and psychological state (e.g., the lacking authenticity of the facial expression). However, robotic stimuli tended to draw attention to salient mechanical body parts (visible in the pelvic area of the machine-like robots), indicating that due to curiosity, there might be a need to visually explore the robotic stimuli more deeply. This might signify both curiosity toward sexualized technologies and difficulties processing the visual information of a human-like machine. All in all, the results of the present study offer a new perspective for the frequently discussed topic of sexualized robots by showing that humanity evokes more deep-rooted processes of visual attention compared to robotic replications of human-like cues. More fundamental research on initial as well as evolving perceptions of sexualized robots is needed in order to gain a more elaborated understanding of underlying psychological processes that are relevant for the interaction with sexualized robots.

## Ethics Statement

This study was carried out in accordance with the recommendations of the ethics committee of the division of Computer Science and Applied Cognitive Sciences at the Faculty of Engineering of the University of Duisburg-Essen (Germany) with written informed consent from all subjects. All subjects gave written informed consent in accordance with the Declaration of Helsinki.

## Author Contributions

JS and NK conceived and designed the experiments. JS performed the experiments, analyzed the data, and wrote the original paper. NK reviewed and edited the paper and supervised.

## Conflict of Interest Statement

The authors declare that the research was conducted in the absence of any commercial or financial relationships that could be construed as a potential conflict of interest.

## Footnotes

1 https://www.apa.org/ethics/code
